# "I feel too lethargic to do physical activity": Perceptions of Iranian adults on the barriers to perform regular physical activity

**DOI:** 10.34172/hpp.2021.60

**Published:** 2021-12-19

**Authors:** Behrouz Fathi, Haidar Nadrian, Mina Hashemiparast, Saeed Nikookheslat, Safooreh Esmaeilzadeh, Rahim Khodayari-Zarnaq

**Affiliations:** ^1^Department of Health Policy and Management, School of Management and Medical Informatics, Tabriz University of Medical Sciences, Tabriz, Iran; ^2^Social Determinants of Health Research Center, Tabriz University of Medical Sciences, Tabriz, Iran; ^3^Department of Health Education and Promotion, School of Public Health, Zanjan University of Medical Sciences, Zanjan, Iran; ^4^Department of Exercise Physiology, School of Physical Education and Sport Sciences, University of Tabriz, Tabriz, Iran; ^5^Department of Health Education and Promotion, Tabriz University of Medical Sciences, Tabriz, Iran; ^6^Tabriz Health Services Management Research Center, Health Management and Safety Promotion Research Institute, Tabriz University of Medical Sciences, Tabriz, Iran

**Keywords:** Physical activity, Barriers, Qualitative research, Middle aged

## Abstract

**Background:** Despite the intention to perform physical activity (PA), a number of individuals cannot manage to have PA program on a regular basis. In this study we explored the barriers of regular PA behavior among healthy adults in Tabriz, Iran.

**Methods:** This qualitative study with a conventional content analysis approach, was carried out from June to September 2020. Nineteen 30-59 years old individuals, were purposefully (purposive sampling) selected to participate in the study. The participants were formerly registered as "physically inactive" in the health records of Tabriz Health System. Individual semi-structured interviews were conducted until data saturation. Data were managed using MAXQDA-10 software.

**Results:** The barriers of regular PA that emerged from our data were being listless and lethargic, non-supportive environment, disintegration in PA education chain, and restrictive social norms.

**Conclusion:** Our findings uncover several PA barriers that are less discussed in the literature. Poor level of regular PA among adults in Iran, as a developing context, is rooted in perceptions with social and economic origins, which should be taken into account by public health policy makers while planning PA promotion programs in such communities. To promote regular PA among healthy adults in developing countries, regular PA programs should be tailored to bridge the gap between their recognition of PA barriers and subsequent behavior change through creating group dynamics highlighting the measures to diminish the behavior.

## Introduction

Positive effects of physical activity (PA) on physical and psychological wellbeing,^1,2^ mortality and morbidity,^1,3,4^ and quality of life are well documented.^2^The economic costs of chronic diseases due to insufficient PA impose a significant economic burden on governments and populations.^2,5,6^ Despite the known benefits of PA, about 31% of adults throughout the world are physically inactive and/or with low levels of PA-based on the definition of the World Health Organization (WHO) (at least 150 min/wk with moderate intensity,^7^ or high-intensity PA for 75 min/wk or a combination of the two).^3^ So, the lack of PA is considered as one of the biggest challenges of the 21st century.^8^

In an Iranian national study conducted in 2020 using the STEPwise Approach to Non-communicable Diseases Risk Factor Surveillance (NCD-STEPS) in people aged 18−64, about 34.5% of adults were reported with low levels of PA.^9^ In 2018, WHO announced the goal of 15% reduction in physical inactivity in adults by 2030.^3^ Current studies, however, show that the goal seems not to be achieved considering the impact of coronavirus disease 2019 (COVID-19) pandemic on further reduction of PA in adults.^10,11^

As middle-aged individuals, adults are biologically and economically the productive age group of all societies, in a way that a wide range of family and community health and development are affected by their health, morbidity and mortality.^12^ Therefore, health promotion and lifestyle modification, including PA promotion, are among the main necessities for this age group.^12^ Understanding the determinants of PA is critical for informing the formulation and implementation of appropriate policies and interventions.^13^ It seems also necessary to have a deep understanding on adult’s experiences about factors associated to PA behavior with the hope to discover thought processes which may affect the occurrence of intended behavior.^14^

Several intrapersonal, interpersonal, economic, social, and cultural challenges are reported that may restrict PA.^5^ Identifying the barriers and factors affecting PA, and understanding the causes of physical inactivity may provide health policy makers and stakeholders with an insight into planning for evidence-aware interventions to promote the behavior.^5,15^

Various studies have been conducted on factors associated to PA, but mostly are cross-sectional with reports only on the associations between different factors and PA.^9^ There is also a scarcity in the studies that explore the reasons for physical inactivity in healthy adults who have behavioral intention to do the behavior. In this regard, an important question bears in mind that why such individual my not perform PA, despite the high level of intention to perform the behavior. Considering the great role of intention in predicting the changes in individuals’ behaviors, it seems important to identify the gap between intention and actual behavior. To help in discovering the barriers of PA, as a complex human behavior with a wide range of influential factors,^14^ conducting qualitative research seems to be useful, as it enables the researcher to explore the phenomenon without any prior preliminary assumption on the associated factors. Therefore, in this study we explored the barriers of regular PA behavior among healthy adults in Tabriz, Iran.

## Materials and Methods

### Study design and participants 

This qualitative study with conventional content analysis approach,^16,17^ was carried out to explore the reasons of inactivity among adults in Tabriz, Iran. The duration of study was from June to September 2020.

Participants were middle-aged individuals who were registered as inactive in the health records of Tabriz Health System. All citizens covered by private and public urban health centers in Tabriz have electronic health records and receive primary healthcare services. The level of performing PA as well as behavioral intention to engage in the behavior (based on the Stage of Change Theory [SCT]) are among the health records of all citizens in the health centers. The first researcher of the study investigated the health records of the population in the health center to identify those inactive individuals who were reported to be at the “contemplation” and/or “preparedness” stages of change for PA, based on SCT. From those healthy adults (aged 30−59 years) with the lowest level of PA, yet with a high level of behavioral intention to engage in the behavior, nineteen individuals (Table 1) were purposefully selected with maximum variation in terms of age, level of education, gender, marital status, and place of residence were invited to participate in the study. Through phone call, the participants were first asked whether they had been physically active for the past 2 weeks [Having daily physical activities such as walking, cycling, and running in a park for at least half an hour for 5 days a week]. If they responded “no”, then they were asked whether they still intend to have a regular PA. Those individuals who responded “yes” to this question were then invited to participate in the study.

**Table 1:** Demographic characteristics of the participants

**Participant**	**Age**	**Gender**	**Education**	**Marital status**	**Occupation**
P1	35	Female	Master	Single	Student
P2	54	Female	Primary school	Married	Housewife
P3	47	Male	Middle school	Married	Self-employment
P4	47	Male	Associate diploma	Married	Municipal employee
P5	41	Male	Diploma	Married	Private company service employee
P6	45	Female	Bachelor’s	Married	Housewife
P7	38	Female	Diploma	Married	Housewife
P8	42	Male	Middle school	Married	Private company employee
P9	46	Female	Bachelor’s	Single	University of medical employee
P10	41	Female	Diploma	Married	Housewife
P11	36	Female	Master	Married	Health center financial employee
P12	51	Female	Diploma	Married	Tractor manufacturing employee
P13	37	Female	Middle school	Married	Housewife
P14	33	Male	Diploma	Married	Self-employment
P15	50	Female	Associate diploma	Married	Retired
P16	50	Male	Diploma	Married	Self-employment
P17	45	Male	Master	Married	Health center employee
P18	40	Male	Bachelor’s	Married	Self-employment
P19	31	Female	Bachelor’s	Divorced	Unemployed

### Data collection

Individual semi-structured interviews were conducted to collect data. Interviews were performed face-to-face using an interview guide (Box 1). The main research question was “How do the middle-aged citizens in Tabriz explain the barriers of PA?” Interview sessions were conducted in an agreed environment and time between the researcher and the participants. Interviews continued until data saturation. At the beginning of each interview session, a brief explanation of the study and its purpose was provided to the participants, and they were then interviewed after ensuring the confidentiality of data. Examples of the main open-ended questions were” How would you explain the level of your PA?”, “What are the barriers for your exercise”? And then some probing questions were asked according to the interview schedule, and the participants’ answers. An example of the probing questions was “Would you please explain more about your statement?” Each interview lasted from 45 minutes to 1 hour, and the interviews were recorded using a voice recorder.

Box 1. **The main interview questions regarding PA in participants**

1. How would you explain the level of your PA? (meaning exercising daily for at least half an hour, 5 days a week so that you feel sweaty and short of breath, such as walking, cycling, or running in the park)?
2. What are the barriers to exercising for you?
3. As a man/woman, in our city, what problems you experienced in exercising?
4. How exercising can be easier for you?
5. As a housewife/worker/man, how can you exercise at home/at work?
6. How many training and sports promotion programs, which have been provided in health care centers are effective in motivating you to exercise?
7. What programs of exercise are you interested in?
8. What is the best approach that motivates you to exercise?

### Data analysis

The interviews were transcribed verbatim immediately after each interview session, and the text was reviewed for accuracy. Conventional content analysis was applied to analyze data. MAXQDA software version10 was used to manage data. All the transcripts were carefully read and re-read, and the initial codes were drawn from the data. Collating the codes into themes, a coding frame was developed in close discussion within the research team members. The first author conducted and analyzed all interviews, and derived the themes from his preconceptions. He kept the research question in mind while conducting and analyzing the interviews. One out of six transcripts were randomly selected, and coded by a second researcher to account for inter-rater reliability.

### Trustworthiness

The criteria outlined by Lincoln and Guba^18^ were applied to ensure the rigority of the study. Member checking was carried out in meetings with the respondents to confirm the preliminary findings. The analytical process was reviewed by colleagues familiar with the qualitative approach. Moreover, the purposeful sampling with a maximum variation approach enhanced the credibility of the data, as it yielded to the participants with a wide range of socioeconomic status, educational attainment, and work experience. Finally, re-checking the analytical codes with the experts in the field of PA strengthened the confirmability and credibility of the data.

## Results

The mean age of participants was 42.5 years and 57 percent were female. The level of education for 63% was diploma and less. About 84% were employed, 26% were housewife, and 26 percent were self-employed.

In total, 1278 codes, seventeen categories and four themes were emerged from the data (Table 2). The reasons for physical inactivity in the middle-aged individuals with high level of intention to perform the behavior were as follow: *Being listless and lethargic*, *non-supportive environment*, *disintegration in education chains*, and *restrictive social norms*.

**Table 2:** Summarizing the main results including main themes and sub-themes

**Main Theme**	**Sub-theme**	**Examples of codes**
Being listless lethargic	Lack of mood and motivation	Laziness and impatience
		lack of motivation and spiritual lethargy
	Being half-hearted and lackluster	Worrying’s about life’s problems
		Individual tensions with others
Non-supportive environment	Financial hardship	Family financial problems
		Economic pressure on society
	Lack of perceived social support	Lack of companions for exercise
		Lack of family support
	poor political and legal support	Lack of incentive and punishment policies
		Lack of allocation of resources and facilities
	Inadequate availability/accessibility to sports spaces and equipment	Inadequate urban and peripheral infrastructure
		Lack of sports facilities and equipment
	Climate conditions, air pollution and seasonal limitations	Long winter season
		Air pollution
	Restrictions of COVID-19 pandemic	Closure of sports venues and activities
		Refusal of people to attend sports venues and clubs
Disintegration in PA education chains	The structural and executive problems of education	Lack of skilled sports trainer in public sports spaces
		Inadequate content of training with people's needs
	poor institutionalization of PA	The symbolism of sports activities in schools
		Lack of priority in sports in schools
	Lack of Physical activity literacy	Unaware of strategies to overcome mental barriers to exercise
		Unaware of the temporary side effects of exercise
	Misunderstanding the necessity of PA	Misconceptions about the effect of PA on the body
		lack of positive personal experiences
Restrictive social norms	Lack of health-oriented lifestyle	Prevalence of unhealthy lifestyle
		Lack of general atmosphere for PA
	Cultural and gender constraints	Limitations resulting from the role of women in the family
		Restrictions on harassment of women in public spaces

### Being listless and lethargic

*Lack of mood and motivation*

As participants reported, they tend to postpone performing PA to another time despite their interest in the sport, and their high intention to do the behavior. They tried to justify their inactivity by stating that laziness and sedentary behaviors are more enjoyable than having PA. Impatience and lethargy, and lack of motivation and spiritual lethargy were the obstacles that impeded them to adopt PA and/or to continue the behavior. A participant said:

*“I used to tell myself that I will go running with my friends in the mornings. But, I cannot wake up in the morning because the morning sleep is much more fascinating than waking up and jogging, and so I get bored either…”*(Participant [p.] 16).

In addition to laziness, several participants noted the role of discouragement in boredom and lack of motivation. One of the participants stated:

“*Another factor is discouragement… being cold feet turns a person into a moving corpse. It makes you like you have no motivation to do anything else, even regardless of exercise, cooking and shopping, you do not spend time even for your children to take care of”*(p. 19).

*Being half-hearted and lackluster*

A majority of participants expressed the tension caused by worries of life as a barrier of performing regular PA. They believed that the tensions of daily life, due to life difficulties in the society and high workload, reduce their level of mood and energy, which consequently impede them from performing PA:

*“The situation in our society is such that one should be worry and stressful about his family and his life. When I have a wide range of problems that worry me, I consequently lose my temper and mood, and when my mood drops, it is hard for me to feel cheerful to pursue what I love, such as having regular PA”*(p. 9).

*“I often do not have time even for myself. After working for 12 to 13 hours a day, I am so tired that I do not have a minimum of energy to think about sports”*(p. 4).

### Non-supportive environment 

*Financial hardship*

Most of participants emphasized the role of economic pressure on families’ financial issues, which, in turn hinders middle-aged individuals, mostly as the breadwinners of the families, to give priority to PA. As they reported, lack of sufficient income in the family prevents the family members from spending money and time on PA.

*“When the level of my welfare and economic facilities is low, no matter how much you encourage me to exercise, it certainly does not affect. The fact is that the financial situations in the current situation of our society are very stressful and no one can spend extra money on exercise”*(p. 3).

*Lack of perceived social support*

Participants believed that the families in the community, as a social and educational institution, do not take action to make PA a habit among the members, and thus PA is not prioritized as a part of daily life plan within families. Lack of companionship while performing PA was also noted as a discouraging factor for continuing the behavior. Moreover, participants believed that a high number of women are not encouraged to have a regular PA plan by their family members and community, and even are not allowed to do PA by their spouse, in many cases.

*“The families do not support the housewife women for having exercise... Supports like assisting in childcare and/or homework”*(p. 12).

*“I like to have someone as a partner while exercising to be enthusiastic. I do not like to go alone. If there is someone to come with me for a walk or gym in the evenings, I will be happy, but I have no one around”*(p. 1).

*Poor political and legal support*

As participants believed, regular PA of adults in the society is not supported by the governance and the stakeholders. They believed on a lack of positive attitude towards staff’s PA among employers and managers in general. As the stakeholders do not give priority to the behavior of government’s staff, they are not concerned about the development of PA infrastructure and policies that facilitate and encourage PA among middle-aged population.

*“Actually, the authorities do not have a positive attitude toward sports. There is no incentive policy in the workplace. How many budget of the organizations and industries is actually spent on personnel PA? Who cares about these issues?”*(p. 9).

*Inadequate availability/accessibility to sports spaces and equipment*

Participants repeatedly complained about the lack of sports facilities in their surroundings, arguing that urban structures, like sidewalks and parks, and gyms were not attractive enough to encourage them to play sports. As they noted, the unsuitable physical and cultural environment of the gyms have made individuals reluctant to attend these places.

*“Sidewalks are often unusable and are often blocked or limited by servicemen. I have also limited access to gyms and my favorite sports. Gym clubs are mostly situated in the underground floors of the buildings with poor lighting and ventilation, and so they are not pleasant.”*(p. 12).

*Climate conditions, air pollution and seasonal limitations*

The long cold season in the city, as a metropolitan area, and air pollution, which results in temperature inversion particularly in cold seasons, were reported as barriers for regular PA among the participants.

*“In our city, when you go out in cold weather, especially when you are getting a little older, you want to exercise, but the effect of humidity and cold weather on your body, and also air pollution is more annoying and disrupts your body system”*(p. 15).

*Restrictions of COVID-19 pandemic*

The *COVID-19*pandemic has led to the closure of gyms and sports activities. Participants also reported the refusal of attending sports clubs and gyms, due to the fear of contracting the disease, and taking care of their school children as a result of school closures. So, the pandemic has limited the participants more to perform regular PA as their time is much occupied by the abovementioned difficulties.

*“Before the outbreak of Coronavirus, I used to take the kids to school and occasionally go to the gym in front of our house, but now I cannot. I prefer not to use the public area. Kids have homework and I do not have time at all”*(p. 13).

### Disintegration in PA education chains 

*The structural and executive problems of education*

Participants’ statements indicated the lack of well-educated PA trainers with sufficient skills and knowledge in the society. As participants reported, the PA training methods used by most trainers are not so attractive, and the content of their trainings does not address the needs of PA trainees. On the other hand, PA education in the social media is not continuous, and is mostly propaganda with only a sensitizing aspect.

*“No one showed the suitable training to me; what exercises and with what intensity is suitable for me as a person with somewhat high blood pressure? Someone should guide you correctly and follow up with you to make PA a habit, someone who knows how to motivate and make push toward PA”*(p. 4).

Another obstacle for the presence of participants in parks was reported to be the lack of PA training programs and PA trainers in the local places like parks, at least in the mornings. A trainer with sufficient skills and expertise in providing movements tailored to the physical condition and needs of participants. Lack of morale and motivation of training in the trainer, lack of sufficient communication skills, poor ethical standards, and inappropriate physique of the trainer were other barriers of attending the parks for performing PA, as reported by participants.

*“There is no trainer in the parks. The gym coach has inappropriate behaviors and/or physique, and has not been educated to train others”*(p. 11).

*“I have never heard or seen a special training program for PA in the park”*(p. 7).

*Poor institutionalization of PA*

Referring to the role of education in institutionalizing the culture of PA in human life, participants stated that PA education activities in schools and the society should be purposefully planned and implemented to spur the individuals to internalize the behavior. However, during school, PA activities and PA education are not given priority, and are presented as an ignorable issue.

*“Since the time of primary school, we have been taught that PA can be easily ignored. We have learned this culture of “PA insignificancy” at schools, and passed it onto the community”*(p. 7).

*Lack of physical activity literacy*

Lack of knowledge and skills to perform regular PA was another reason reported by participants. Participants believed that a majority of people in the society do not have enough knowledge about the benefits of PA, the side effects of a sedentary lifestyle, the simple strategies of adopting regular PA at home and/or in an apartment, and strategies for overcoming mental barriers of PA.

*“We are illiterate about PA, and we do not know the obligations. For example, I do not know how to exercise inside my apartment so that the noise not to disturb the neighbor”*(p. 13).

*Misunderstanding the necessity of PA*

As a majority of participants reported, a majority of people consider PA as a remedy for those who are diagnosed with a chronic disease. They believed that PA is not a priority in their life as they are healthy individuals with no chronic disease and/or obesity, and their associated symptoms.

*“Fortunately, I do not have any chronic illness, nor I am overweight that motivates me to control it by PA…. So, the pleasure and health effects of exercising have not come into my mind, so exercise is my last priority”*(p. 11).

### Restrictive social norms

*Lack of health-oriented lifestyle*

Participants believed that the current general atmosphere of community do not stimulate regular PA. The mechanization of lifestyle and dense and crowded spaces has tended the people to sedentary behaviors and unhealthy personal habits. Some participants confessed that they do not value health, and do not care about regular PA until they became ill.

*“When we leave our houses to go to work, we go and return by car. Today, our lives have shifted to apartment living and sedentary jobs. There is also the limitation of the physical space, and it is not possible to do many exercises inside an apartment”*(p. 5).

*Cultural and gender constraints*

The unsuitable cultural atmosphere of the parks for women’s PA, and lack of a calm and safe environment for them, as well as negative subjective norms on exercise clothing were the obstacles to perform regular PA in open and public spaces, as reported by female participants. Participants believed that women exercising in public places, and even in gyms, is something that is not fully established in the culture of the people, and is not still accepted as a social norm, particularly among the people with traditional culture. Participants also reported that women mostly in the community prefer to spend more time on their motherhood and housewife duties than caring for themselves and doing regular PA, which may be originated from their traditional culture.

*“I am in the lower part of the city, there is a negative attitude towards a woman who goes to exercise, or takes a sports bag and goes to a gym. Women’s job is to sit at home, cook, and do housekeeping. I would like to go biking, but it is not allowed for women”*(p. 19).

## Discussion

In this study, we explored the barriers of regular PA behavior among healthy middle-aged people in Tabriz, Iran. To our knowledge, this was the first qualitative study that explored the reasons for physical inactivity in middle-aged people with high behavioral intention.^19,20^ According to our findings, *Being listless and lethargic*, *non-supportive environment*, *disintegration in education chains*, and *restrictive social norms*. Lack of mood and motivation reduce individual’s vitality and liveliness, which make them unable to overcome the obstacles, and thus reluctant of performing PA.^21-23^ According to Teixeira, motivation is a fundamental factor for supporting continuous PA, which is associated with important health outcomes.^24^

On the other hand, tensions caused by worries of daily life may reduce the levels of mood and energy, which may in turn result in a chronic fatigue that discourage people from performing PA.^25^

People’s misunderstandings and/or wrong beliefs about the effects of PA, and the lack of positive personal experiences of participants separate them from PA. According to Azjen, behavioral beliefs are a driving force towards a particular attitude, and are determined by perceived outcomes of performing the behavior and people’s evaluation of any possible consequences.^26^ People may believe that PA improves their health. However, they may also find it time-consuming and tiresome. So, what ultimately constitutes people’s attitudes and behaviors is the dominance of positive beliefs over the negative ones. In the present study, it seems that misunderstanding on the necessity of PA among participants have overcome their positive beliefs, which may impede them to perform regular PA. So, having an emphasis on resolving and handling the misunderstandings on the necessity of the behavior, and highlighting its true benefits can help people in making knowledgeable decisions on their regular PA program.^27^

The second factor is the urgent daily needs of human beings. Because of family and economic problems, participants prefer to take time for their urgent needs instead of PA. Excessive work and financial hardship, in addition to the role of family,^28,29^ make them unable to include PA in their daily routine. On the other hand, lack of time is one of the important barriers to not doing PA. Lack of time has a negative effect on PA.^28^ However, participants’ statements were mostly indicated the inability to manage time, which leads to a lack of regular PA. The majority of participants also emphasized the role of families’ financial problems and the economic pressure on society, as a serious obstacle to PA. There is a clear correlation between economic and social status and PA in low- and middle-income countries.^5^ Previous studies have reported that more financial stress is associated with fewer PA in individuals.^30^

From the participant’s point of view, another obstacle for regular PA was non-supportive environment, such as financial hardship, restrictions caused by COVID-19,^10,11^ lack of perceived social support, poor political and legal support, inadequate availability and accessibility to sports equipment and spaces, climate conditions, air pollution and seasonal limitations that can have an synergistic effect on each other.^31,32^ This means that behavior does not occur on its own, and individuals do behave in response to environmental conditions.^31^ In this regard, participants believed that a majority of families in the community does not play their role well, as educational and social institutions, in building regular PA culture in its members, and thus PA has not become part of daily life plan among families. As an instance, participants stated that most of mothers are not encouraged to have regular PA by their family members and the community. This finding is similar to the findings of several previous studies that reported associations between social support and regular PA.^33,34^ In the case of social and family support, the of lack of time, as a mainly reported barrier of regular PA can also be overcome.^21^ As Bandura stated, the observation of efforts and success in peers might strengthen people’s beliefs, and lead them to become familiar with their capabilities of doing the same activity which may result in achieving a higher level of success.^35^ In our study, it seems that the absence of family and social support has made a negative impact on individuals’ beliefs on their capabilities, and thus impede the conversion of behavioral intention into PA habit.

Our findings showed the belief of participants on low levels of political and legal support for PA in various groups of the population, due to the lack of managerial and political support to promote regular PA. As they reported, due to a lack of positive attitude towards the behavior among employers and managers, the policy of regular PA promotion is not considered in the list of their priorities. Consequently, there is insufficient attempt to develop PA infrastructure, allocate resources, and adopt and implement policies to encourage regular PA. Meanwhile, policy interventions can affect all populations for a long time.^5^ Policies can also supply guidelines for collective and/or individual behavior, which can be done as either formal or informal legislation and oversight by governmental and non-governmental organizations.^36^ As policies are an integral part of PA planning, resource allocation and public health regulation (e.g., environmental policies and urban design standards),^37,38^ our findings can serve as a basis for building community-centered policy options aiming at regular PA promotion programs within communities.

In our study, participants frequently complained about the lack of sports facilities in their surroundings, and believed that urban structures and clubs do not have the required attractiveness and capacity to encourage them to perform regular PA. Adequate availability and accessibility to sports equipment and venues (e.g. neighborhood-built environment), and paying attention to their aesthetic aspects can increase the desire and willingness to participate in PA activities.^33,39-41^ It seems that creating a positive social atmosphere in the community can influence the promotion of PA among other people within the society.^42^ On the other side, the lockdowns and restrictions of sports venues and activities due to COVID-19 pandemic have further fueled the situation.^10,11^ Moreover, the geographical location of the Tabriz metropolitan area, the long cold season, and also the air pollution of the city have all impacted negatively on performing regular PA, and impeded the use of sports facilities in the parks, as participant reported. PA behavior seems to be a function of weather conditions and seasonal changes. For example, people tend to have a higher level of sedentary behaviors in cold, rainy, and cloudy days, and also on shorter days (photoperiod) and the time of air pollution.^43,44^

A majority of participants stressed a disintegration in the education chain of regular PA. Similar to those identified in the present study, the appropriateness of PA educational content, characteristics of the PA programs, the communication skills of the educators, and taking into account the participants’ educational needs had a positive effect on participants’ satisfaction and regular PA adoption.^45^ Our participants pointed out to the role of education in institutionalizing the culture of PA in lifespan, while during school, PA programs are not given a priority, as they reported. In consequence, the lack of institutionalization of PA during the school time of the middle-aged people has now resulted in a low level of PA literacy among them. Whitehead defines PA literacy as the “motivation, confidence, physical competence, knowledge, and understanding to value and take responsibility for engagement in PAs in life”.^46^ PA literacy can be a promising strategy for achieving lifelong participation in PA, and is a meaningful approach to reducing sedentary behaviors and preventing chronic diseases.^47^

From the participants’ point of view, the current general atmosphere of the community is not PA-friendly, and might not motivate people to perform PA. They believed that urbanization and mechanization can lead to laziness and increased sedentary behavior, through changing the patterns of work, transportation, entertainment, and consumption. This finding was similar with those reported in a previous study.^48^ On the other hand, due to the competitive demand of job responsibilities and workload and also family commitments, the middle-aged people seem to not give priority to PA, despite its beneficial effects on their health.^24^ Such factors, therefore, may hamper the people from turning intentional PA behavior into a habit.

Cultural and gender constraints, including restricting roles of women in the family, harassment of women in public spaces, women’s clothing in public spaces, and time and space constraints were identified as the factors that may hinder women’s PA in public settings. According to Smith, cultural beliefs, social isolation, and the insecure environment in the neighborhood can be sociocultural barriers to women’s access to recommended levels of PA.^33,34^

## Conclusion

Our findings uncover several PA barriers that are less discussed in the literature. Poor level of regular PA among adults in Iran, as a developing context, is rooted in the perceptions with social and economic origins, which should be taken into account by public health policy makers while planning PA promotion programs in such communities.

To promote regular PA among healthy adults in developing countries, regular PA programs (at the practice and maintenance stages) should be tailored to bridge the gap between their recognition of PA barriers and subsequent behavior change through creating group dynamics highlighting the measures to diminish the behavior.

## Acknowledgements

Special thanks to Tabriz University of Medical Sciences and Tabriz Health Center for their supporting.

## Funding

This study was part of a PhD dissertation, supervised by RKH-Z and HN supported by Tabriz University of Medical Sciences (Grant No: 63536)

## Competing Interests

Haidar Nadrian is an Associate Editor in Health Promotion Perspectives. The other authors declare that there is no conflict of interest.

## Ethical approval

This study was approved by the regional ethics committee of Tabriz University of Medical Science; Approval ID: IR.TBZMED.REC.1398.569.

## Authors’ Contributions

BF: methodology, data acquisition, analysis, writing – original draft, writing – review & editing, project administration. HN: methodology, writing – review & editing, supervision. MH: writing – review & editing. SN: data acquisition, writing – review & editing. SE, RKZ: methodology, analysis, data, writing – review & editing, supervision. All authors gave final approval to the version to be published, and agree to be accountable for all aspects of the work.

## Disclaimer

The authors claim that no part of this paper is copied from other sources.
